# Corneal confocal microscopy detects small fibre neurodegeneration in Parkinson's disease using automated analysis

**DOI:** 10.1038/s41598-020-76768-x

**Published:** 2020-11-19

**Authors:** Sze Hway Lim, Maryam Ferdousi, Alise Kalteniece, Lewis Kass-Iliyya, Ioannis N. Petropoulos, Rayaz A. Malik, Christopher Kobylecki, Monty Silverdale

**Affiliations:** 1 Department of Neurology, Salford Royal NHS Foundation Trust, Manchester Academic Health Sciences Centre, University of Manchester, Manchester, UK; 2 Faculty of Biology, Medicine and Health, Division of Cardiovascular Sciences, University of Manchester, Manchester, UK; 3 Weill Cornell Medicine-Qatar, Qatar Foundation-Education City, PO Box 24144, Doha, Qatar

**Keywords:** Biomarkers, Neurology

## Abstract

We studied the utility of corneal confocal microscopy (CCM) in detecting a reduction in corneal nerve parameters in a large cohort of patients with Parkinson’s disease (PD) compared to controls using a fully automated potentially scalable method of analysis. We also assessed if CCM parameters are related to the severity and sub-type of PD. 98 participants with PD and 26 healthy controls underwent CCM with automated corneal nerve quantification**,** MDS-UPDRS III, Hoehn and Yahr scale, Montreal Cognitive Assessment, Parkinson’s Disease Questionnaire-39 and PD subtype assessment. Corneal nerve fibre density (mean difference: − 5.00 no/mm^2^, 95% confidence interval (CI) [− 7.89, − 2.12], *p* = 0.001), corneal nerve branch density (mean difference: − 10.71 no/mm^2^, 95% CI [− 16.93, − 4.48], *p* = 0.003), corneal total branch density (mean difference: − 14.75 no/mm^2^, 95% CI [− 23.58, − 5.92], *p* = 0.002), and corneal nerve fibre length (mean difference: − 2.57 mm/mm^2^, 95% CI [− 4.02, − 1.12], *p* = 0.001) were significantly lower in PD participants compared to controls. There was no correlation between corneal nerve parameters and duration, severity or subtype of PD, cognitive function or quality of life. CCM with automated corneal nerve analysis identifies nerve fibre damage and may act as a biomarker for neurodegeneration in PD.

## Introduction

Traditionally, Parkinson’s disease was thought to be a motor disorder caused primarily by degeneration of the dopaminergic nigrostriatal pathway, but it is now increasingly viewed as a multisystem disease secondary to widespread deposition of alpha-synuclein^1^. Indeed, there is substantial neuropathological evidence of Lewy bodies in both central extra-nigral and peripheral nervous system structures^2–5^. Phosphorylated alpha-synuclein has been demonstrated in autonomic nerves of the colon^4^, cardiac plexus^5^ and cutaneous c-fibres^2^. This departure from the basal ganglia-centric model of PD, allows us to explore the utility of peripheral nerve damage as a biomarker in PD.

The heterogenous clinical features and different rates of progression in PD suggests that there may be distinct subtypes under the umbrella of a PD diagnosis. Research on peripheral nerve involvement in PD is improving our understanding of the pathological mechanisms in PD and enabling better stratification of disease subtypes for prognostication of disease progression. Peripheral neuropathy^6^ and autonomic involvement^7^ in PD have been associated with faster disease progression, as well as certain clinical subtypes such as postural instability/gait disturbance^8^. Small fibre neuropathy has traditionally been studied using skin biopsies and skin biopsies in people with PD demonstrate alpha-synuclein deposition and small nerve fibre degeneration^2,3,9^. However, skin biopsy is an invasive procedure, requiring manual tissue processing and expertise for quantification, which is time consuming. The development of less invasive methods for assessing small fibre neuropathy is crucial for widespread clinical and research use. Corneal confocal microscopy (CCM) is a non-invasive ophthalmic imaging technique that can visualise corneal sub-basal nerve fibres in vivo^10^ and has been used to identify small fibre damage in a range of peripheral neuropathies including diabetic neuropathy^11^, idiopathic small fibre neuropathy^12^, Fabry’s disease^13^ and Charcot Marie Tooth disease^14^.

To date three studies utilising CCM in small cohorts of PD participants have shown small fibre damage in PD participants compared to controls^9,15,16^. We previously showed a reduction in corneal nerve fibre density which correlated with the severity of autonomic dysfunction and motor severity of PD^9^. Podgorny et al. demonstrated a reduction in corneal nerve fibre length and branch density in PD participants compared to controls^15^. Misra et al. demonstrated a reduction in corneal nerve fibre length in PD participants with more advanced PD (Hoehn Yahr stage III and IV)^16^.

In this study we have utilised automated analysis to objectively compare corneal nerve parameters in PD participants to healthy controls and the association between CCM measures and clinical parameters in the PD cohort.

## Methods

### Ethics

NRES Committee/North West approved the pilot (Ref no 12/NW/0086) and larger (Ref no 17/NW/0144) study. Written informed consent was obtained from every participant. This research adhered to the tenets of the Declaration of Helsinki for clinical research involving human subjects.

### Subjects

Patients with Parkinson’s disease aged between 18 and 90 years, fulfilling Queen Square Brain Bank Criteria^17^ were recruited from neurology clinics across Greater Manchester and via Fox Trial Finder and Parkinson’s UK websites between September 2017 and September 2018. Key exclusion criteria included concurrent diagnoses of diabetes, active malignancy, hepatic disease, any other known cause of neuropathy, chronic corneal pathologies, history of refractive surgery and any systemic disease known to affect the cornea such as Fabry’s disease, chronic kidney disease, and Sjogren’s disease. Eighty-four PD participants were screened. Five participants were excluded due to abnormal blood tests suggestive of other causes of neuropathy, two were excluded as they had normal DAT scans, one was unable to undergo CCM and one participant had been concurrently enrolled in a disease modifying drug trial. Seventy-five participants were enrolled into the study in addition to twenty-three PD participants from our pilot study using the same key inclusion and exclusion criteria. Twenty-six healthy age matched volunteers were used as controls. Written informed consent was obtained from all participants.

### Medical history and demographics

Subjects’ gender, age, other medical conditions, medications including dopaminergic therapies, alcohol intake and smoking history was recorded. Duration of disease was calculated from the date of diagnosis to date of assessment. A full blood count, urea and electrolytes, glycated haemoglobin, immunofluorescence anti-nuclear antibodies, B12, Folate, immunoglobulins, serum electrophoresis and thyroid function tests were performed to exclude other known aetiologies of neuropathy.

### Neurological assessment

A clinical examination to detect evidence of peripheral neuropathy was carried out. Movement Disorder Society Unified Parkinson’s Rating Scale part III (MDS-UPDRS III) was used to assess motor severity in the ‘ON’ state. PD participants in the pilot study were assessed in the ‘ON’ state using the Unified Parkinson’s Disease Rating Scale part III (UPDRS III) and the data was converted to MDS-UPDRS III using the conversion formula described by Goetz et al^18^. PD stage was determined using the Hoehn and Yahr scale. Participants recruited between September 2017-September 2018 also had cognition assessed using the Montreal Cognitive Assessment (MoCA) scale and were asked to complete a PDQ-39 questionnaire to assess health related quality of life in PD. PDQ39 summary index (PDQ39 SI), a validated summary score that is derived from the eight-dimension scores gained from the PDQ-39 questionnaire, was calculated for each PD participant.

### Subtypes

Patients recruited between September 2017-September 2018 were subtyped into tremor dominant and postural instability with gait disturbance, based on the ratio of MDS-UPDRS scores as described by Stebbins et al^19^.

### Ophthalmic assessment

Participants underwent a comprehensive ophthalmic assessment by trained optometrists. Both eyes were assessed initially using a slit lamp biomicroscope (Slit Lamp BD 900, Haag Streit) to exclude pathology in the anterior segment of the eye. Corneal confocal images were acquired using a laser scanning corneal confocal microscope: Heidelberg Retinal Tomograph III Rostock Cornea Module (HRT III RCM); Heidelberg Engineering GmbH, Heidelberg, Germany. A × 63 objective lens was used. The field of view was 400 × 400 μm. 2-dimensional images measuring 384 × 384 µm and 10 µm per pixel optical resolution were created.

During the examination, head/chin frames were used to help stabilize the position of the participant’s head. The alignment of the participant’s eyes was maintained by asking the participant to fixate on a white light with the eye contralateral to the one being examined. In addition, a charged couple device (CCD) camera was used to monitor the exact location of the camera on the corneal surface during the examination. Several images were taken from the central cornea of each eye and six images (three per eye) were selected based on standardised criteria^20^.

### Corneal nerve quantification

CCM images were analysed using fully automated software ACCMetrics (M.A. Dabbah, Imaging Science, The University of Manchester, 2010)^21,22^. Corneal nerve fibre density (CNFD): number of main nerve fibres per frame (no/mm^2^), corneal nerve branch density (CNBD): number of intersections between main nerves and secondary nerves per frame (no/mm^2^), corneal total branch density (CTBD): the total number of branch points per frame (no/mm^2^) and corneal nerve fibre length (CNFL): the total length of all nerve fibres per frame (mm/mm^2^) were quantified and a mean was derived for each parameter.

### Statistical analysis

Based on our published pilot study we calculated that at least 80 participants with PD were required (4:1 split, 64 PD, 16 control) with an alpha error of 0.05 and a beta error of 0.8 to demonstrate a difference in corneal nerve metrics between patients with PD and controls. A larger PD group was recruited to enable stratification of the PD participants into different Hoehn Yahr stages, disease subtypes and cognitive status.

IBM SPSS version 25 was used to analyse the results. Chi square test was used to assess for a statistical difference between categorical data. Normality of distribution was assessed by the Shapiro–Wilk test. Independent samples t-test was used to compare means of normally distributed data and the Mann–Whitney U test was used for non-parametric data. Cohen d was calculated to measure effect size: d = 0.2 (small), d = 0.5 (medium), d = 0.8 (large). Two tailed Spearman’s correlation was used to ascertain relationships between continuous variables. One-way ANOVA was used to compare means between groups.

## Results

### Study population

Ninety-eight PD participants were compared with twenty-six controls. The demographics and clinical characteristics of PD participants and controls are shown in Table 1.

**Table 1:** Demographics and clinical characteristics of Parkinson's disease patients and controls.

	PD patients (n = 98)	Controls (n = 26)	*p* value
Gender	28F 70M	10F 16M	0.33
Age (years)	64.0 ± 0.82 (42–81)	62.0 ± 1.40 (49–76)	0.24
MDS-UPDRS III	29.0 ± 1.18 (7–65)		
Disease duration (months)	58.0 ± 4.8 (2–249)		
Hoehn and Yahr stage	I:21 II:63 III:14		
MoCA (n = 77)	26.0 ± 0.3 (17–30)		

Data shown as mean ± SEM (range). PD: Parkinson’s disease, MDS UPDRS III: movement disorder society unified Parkinson’s disease rating scale part III, MoCA: montreal cognitive assessment.

### Corneal nerve morphology in PD participants compared to controls

**Figure 1:**
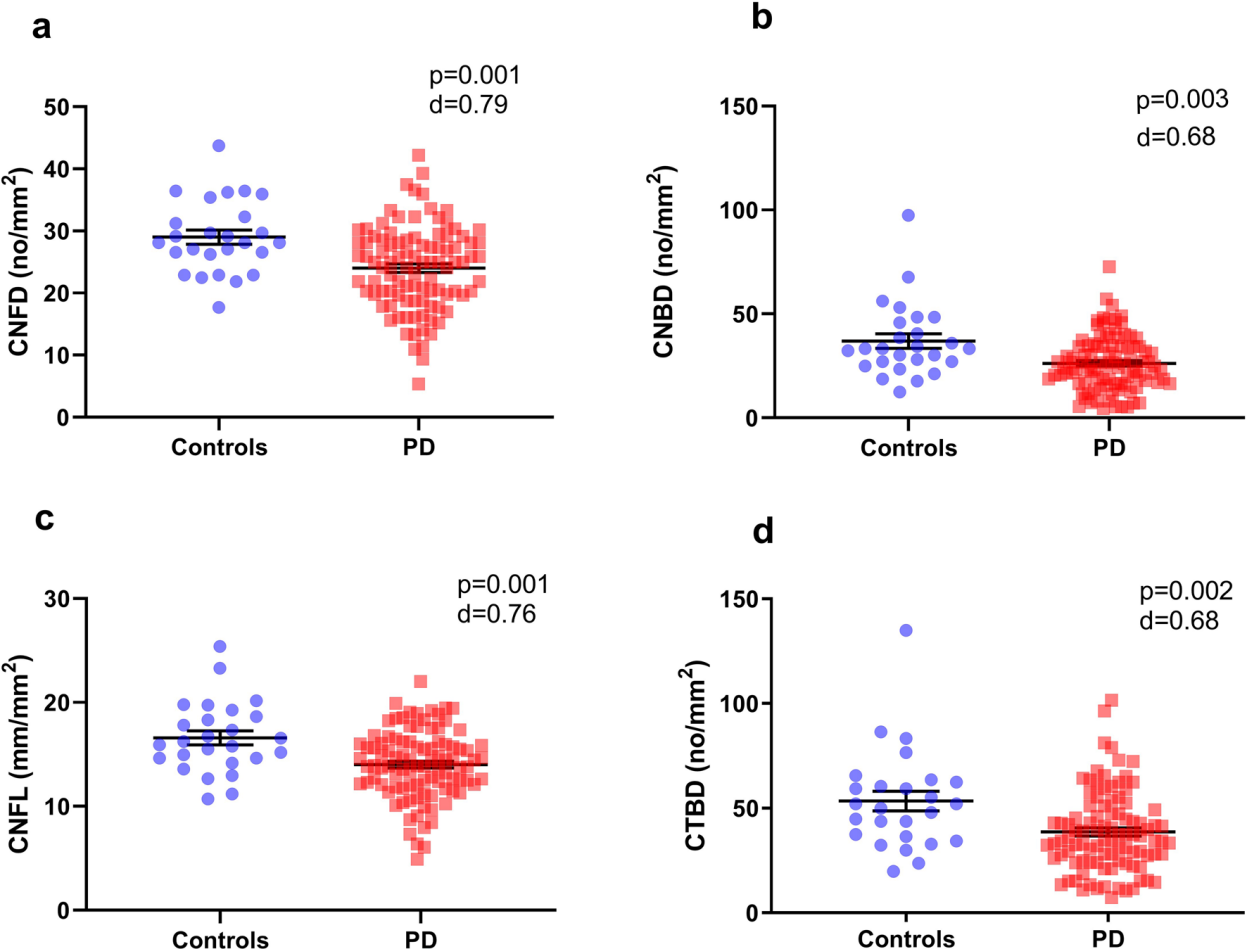
Mean ± SEM of corneal nerve fibre density (CNFD), corneal nerve branch density (CNBD), corneal nerve fibre length (CNFL) and corneal nerve total branch density (CTBD) in patients with Parkinson’s disease compared to controls with significant levels and Cohen d effect size*.*

**Figure 2:**
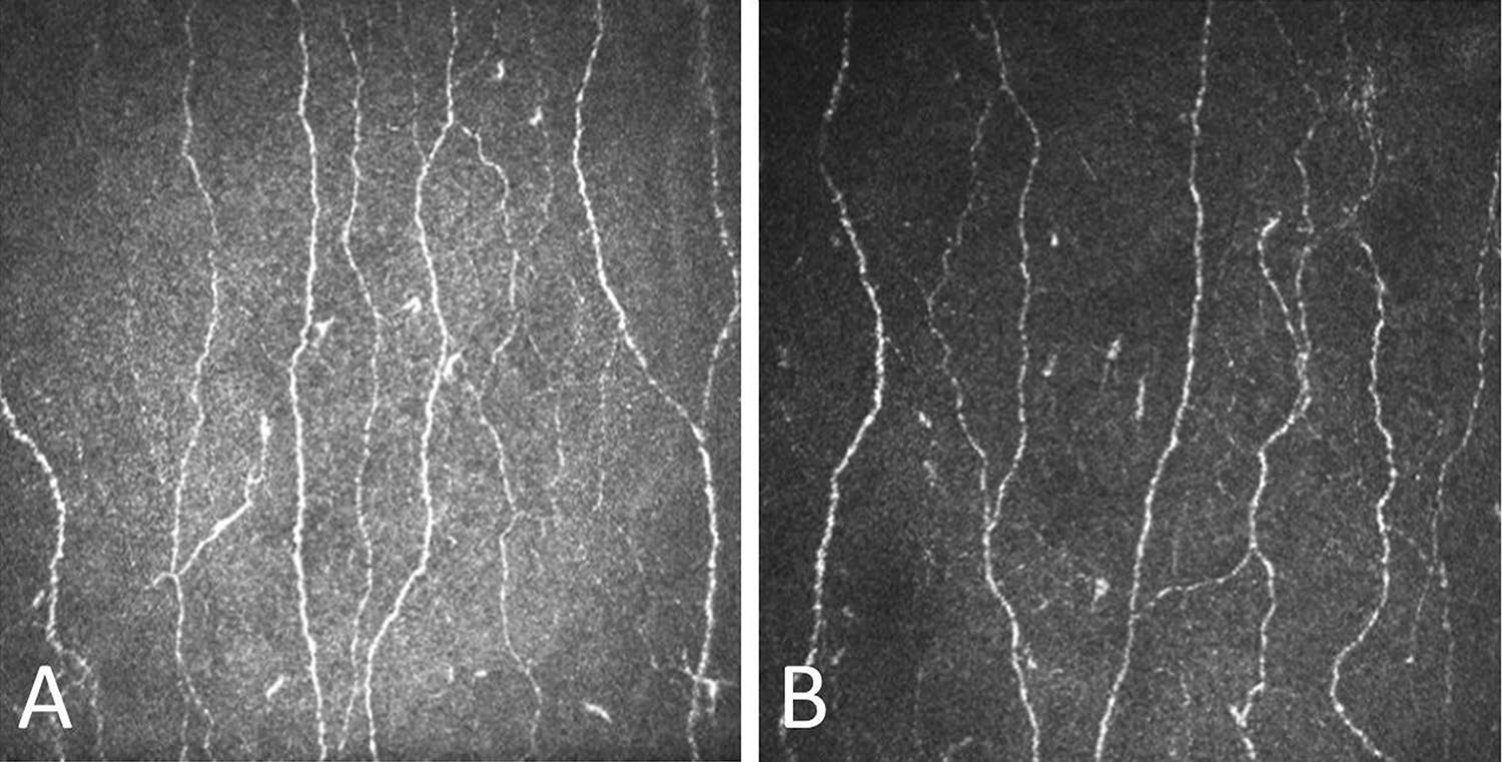
Corneal confocal image of a healthy control (A) compared to a patient with Parkinson’s disease (B) showing an overall reduction in corneal nerve fibre density, corneal nerve branch density, corneal nerve total branch density and corneal nerve fibre length.

The quality of the images acquired for the PD participants was comparable to the controls as patients were assessed in the ‘ON’ state to minimize fatigue and interference from motor symptoms. CNFD, CNBD, CTBD and CNFL were significantly lower in participants with PD compared to controls (CNFD mean difference: − 5.00 no/mm^2^, 95% confidence interval (CI) [− 7.89, − 2.12], d = 0.79; CNBD mean difference: − 10.71 no/mm^2^, 95% CI [− 16.93, − 4.48], d = 0.68; CTBD mean difference: − 14.75 no/mm^2^, 95% CI [− 23.58, − 5.92], d = 0.68 and CNFL mean difference: − 2.57 mm/mm^2^, 95% CI [− 4.02, − 1.12], d = 0.76) (Fig. 1, 2).

### Corneal nerve morphology in PD subtypes

There was no significant difference in CCM parameters between tremor dominant (TD) (n = 42), postural instability, gait difficulty (PIGD)(n = 25) and indeterminate (n = 8) subtypes of PD (Table 2).

**Table 2:** Corneal confocal microscopy parameters in postural instability/gait disturbance vs tremor dominant patients with Parkinson’s disease.

CCM parameter	PIGD (n = 25)	TD (n = 42)	*p* value
CNFD (no/mm^2^)	25.33 ± 1.49	22.57 ± 1.02	0.12
CNBD (no/mm^2^)	27.57 ± 3.27	23.33 ± 1.64	0.20
CNFL (mm/mm^2^)	14.54 ± 0.75	13.39 ± 0.48	0.18
CTBD (no/mm^2^)	38.51 ± 4.65	34.51 ± 2.16	0.93

Data shown as mean ± SEM. CCM: corneal confocal microscopy, PIGD: postural instability/gait disturbance, TD: tremor dominant, CNFD: corneal nerve fibre density, CNBD: corneal nerve branch density, CNFL: corneal nerve fibre length, CTBD: corneal total branch density.

### Corneal nerve morphology between different Hoehn Yahr stages

Although patients in Hoehn Yahr stage II and III had lower values of CCM parameters compared to stage I (Table 3), one-way ANOVA did not demonstrate a significant difference in CNFD (F = 0.218, *p* = 0.804), CNBD (F = 0.792, *p* = 0.456), CNFL (F = 0.448, *p* = 0.641) and CTBD (F = 0.790, *p* = 0.457) between the different Hoehn Yahr stages.

**Table 3:** CCM parameters in patients Hoehn Yahr stage I, II and III.

	Hoehn Yahr	Hoehn Yahr	Hoehn Yahr
	Stage I (n = 21)	Stage 2 (n = 63)	Stage 3 (n = 14)
CNFD (no/mm^2^)	24.88 ± 1.35	23.77 ± 0.88	23.75 ± 1.88
CNBD (no/mm^2^)	29.21 ± 2.82	25.08 ± 1.67	26.94 ± 3.65
CNFL (mm/mm^2^)	14.61 ± 0.69	13.88 ± 0.43	13.72 ± 0.87
CTBD (no/mm^2^)	43.09 ± 3.92	37.04 ± 2.46	39.29 ± 5.23

Data reported as mean ± SEM. CNFD: corneal nerve fibre density, CNBD: corneal nerve branch density, CNFL: corneal nerve fibre length, CTBD: corneal total branch density.

### Correlation between corneal nerve morphology and clinical data

Ninety-eight participants with PD were included in the analysis for correlation between corneal nerve measures, duration of disease and MDS UPDRS III. Seventy-five participants were analysed for correlation between corneal nerve measures with MoCA and PDQ39 SI. MDS-UPDRS, duration of disease, MoCA and PDQ39 SI were not normally distributed. There were no statistically significant correlations between corneal nerve measures and clinical measures of PD (Table 4).

**Table 4:** Correlation between corneal nerve measures, duration of disease, MDS UPDRS III, MoCA and PDQ39-SI.

CCM parameter	Duration of disease	MDS-UPDRS III ‘ON’	MoCA	PDQ39 SI
CNFD	0.024	− 0.109	0.202	0.010
CNBD	− 0.041	− 0.087	0.195	− 0.054
CNFL	0.006	− 0.098	0.184	− 0.045
CTBD	− 0.059	− 0.041	0.179	− 0.127

Data reported as Spearman’s correlation coefficient. No correlations were significant (*p* > 0.05). CCM: corneal confocal microscopy, MDS UPDRS III: movement disorder society unified Parkinson’s disease rating scale part III, MoCA: montreal cognitive assessment, PDQ39 SI: Parkinson’s disease questionnaire-39 summary index.

## Discussion

This study shows a significant reduction in corneal nerve parameters in participants with PD compared to controls. Three previous studies using the Heidelberg HRTIII CCM demonstrated a loss of corneal nerves in patients with PD^9,15,16^. However, the cohorts studied were relatively small and different methods of corneal nerve quantification were used. Although image selection has been shown to be reproducible between investigators^20^, quantification of corneal nerves can vary according to the protocol used for image selection^23^ and whether the analysis is manual, semi-automated or fully automated^24^. Manual analysis is labour intensive, subject to inter/intra-rater variability and requires training to limit variability. Fully-automated CCM analysis using ACCMetrics (M.A. Dabbah, Imaging Science, The University of Manchester, 2010)^21,22^ has been successfully used by our group and others in several previous studies to demonstrate small fibre degeneration^11,15,25^. It has the advantage of being reproducible and the images can be rapidly analysed^21^, enabling scalability of the technique. Here, we use the same fully automated analysis to quantify corneal sub basal nerve parameters in a large cohort of PD patients compared to controls.

Previously we undertook manual corneal nerve quantification and showed a reduction in CNFD but increased CNBD and CNFL in PD patients with relatively mild PD, suggestive of proximal corneal nerve degeneration with more distal regeneration^9^. Misra et al. showed reduced CNFL in patients with more advanced PD^16^, whilst Podgorny et al. showed a significant reduction in CNFL and CNBD, indicative of distal degeneration in participants with recently diagnosed PD^15^. Arrigo et al. demonstrated increased corneal nerve tortuosity and beading and altered trigeminal nerve diffusion on MRI in participants with PD^26^.

In the present cohort of PD participants, we show a global reduction in all corneal nerve parameters using automated analysis. Disease duration and severity as well as the method of analysis can affect the outcome when evaluating corneal nerve degeneration in PD. Nerve degeneration and regeneration is a dynamic process. A previous study by Nolano et al. showed that cutaneous nerve regeneration may accompany degeneration early on in the disease process, but may become less efficient as PD progresses^27^. We have also recently shown evidence of intraepidermal nerve fibre degeneration with impaired regeneration, which correlated to disease severity in PD^28^. Regeneration may cause the number of branches and the total length of nerves to vary depending on stage of disease which may explain the variations in CNFL and CNBD seen between the different studies. Despite the differences in method of analysis between our current study and our previous study^9^, CNFD is consistently reduced in PD patients compared to controls.

Parkinson’s disease is a widely heterogenous disorder. Identifying subtypes and features that determine faster rates of progression is a high priority clinical and research area. Subtyping patients based on motor symptoms was one of the initial methods used to describe different phenotypes in PD. In this analysis, we explored whether there were any differences in CCM parameters between motor subtypes, as PD patients presenting with tremor dominant symptoms are thought to have a more benign course of disease and slower rate of progression compared to the PIGD subtype^29^. There were no significant differences in CCM parameters between different subtypes classified by motor symptoms. The difference in rate of progression between the motor subtypes may not be caused by the overall extent of neurodegeneration as there is evidence that there is differential involvement of neurotransmitter systems and brain structures between the subtypes^30^ Pathological studies demonstrate less cell loss in the substantia nigra pars compacta and the locus coeruleus, with more cell loss in the retrorubral area of the midbrain in patients with tremor dominant compared to non-tremor dominant PD^31^. Motor subtyping has also been criticised for confounding by disease stage and inconsistent reliability as patients with initial tremor dominant symptoms can switch subtypes in later stages and vice versa^32^.

As our understanding of PD has progressed, several studies have shown that non-motor symptoms and involvement of the peripheral nervous system^6,33^ provide additional prognostic value beyond the traditional tremor vs PIGD subtyping. This study demonstrates that some PD patients have fairly marked corneal nerve degeneration whereas others have CCM parameters within the normative range for healthy subjects. It has recently been proposed that there are two separate forms of PD: A peripheral onset form associated with marked autonomic neuropathy prior to involvement of the dopaminergic system and a central onset form with dopaminergic dysfunction preceding autonomic neuropathy^34^. Peripheral neuropathy is associated with a more severe Parkinson’s phenotype^6^. Small fibre damage in the form of autonomic dysfunction such as orthostatic hypotension, constipation, sweating abnormalities and erectile dysfunction in males has also been associated with more rapid disease progression and shorter survival^7^. Thus, earlier identification of a peripheral onset form of PD may enable the identification of a ‘fast progressors’ cohort.

Whilst there was an overall trend for a reduction in CCM parameters with higher Hoehn Yahr stages, this was not significant. This may be because the Hoehn Yahr scale is a categorical scale that describes clinical status which is weighted heavily towards postural instability. Each increment on the scale does not necessarily represent a higher degree of overall disability and non-motor features are not captured by the scale^35^. There can also be a large variation of impairment severities within each Hoehn Yahr category^35^. The Hoehn Yahr scale may not be a sufficiently nuanced scale to tease out the association between corneal nerve changes and features of severe disease phenotype.

We have found no correlation between CCM parameters and disease duration, severity of motor or cognitive impairment, or quality of life. However, motor score or quality of life at a single time point are not in themselves markers of severe PD phenotypes as the scores can be confounded by stage of disease, age and intra-rater variability. The lack of association between CCM parameters and MoCA scores is likely because the PD participants in this cohort had minimal cognitive impairment as demonstrated by the high MoCA scores. Small fibre degeneration may be more closely related to different disease phenotypes i.e. those with and without small fibre degeneration as opposed to disease severity^9^. These participants are being followed up longitudinally to determine how CCM parameters change and correlate with clinical changes over time.

This study was not designed to assess the utility of CCM as a biomarker in PD as a cross sectional study does not enable CCM changes to be monitored and compared to clinical progression. MDS-UPDRS III was also assessed in the ‘ON’ state which may have affected the interpretation of motor severity scores. The assessments were done in the ‘ON’ state to enable the acquisition of high quality CCM images. Autonomic function was not assessed in this study. However, we have previously demonstrated a correlation between autonomic dysfunction and CCM parameters^9^. We were able to achieve our primary objective which was to show differences in CCM parameters using automated analysis in PD participants compared to healthy controls, with significant effect size.

Due to the overlap in CCM parameters between healthy controls and PD patients, CCM cannot be considered to be a diagnostic tool for PD. Nevertheless, the study demonstrates that automated CCM can detect small nerve fibre degeneration in PD. Longitudinal assessment of this cohort may help to define whether CCM allows objective identification of a ‘fast progressor’ cohort and / or an objective measure of disease progression in PD. Further longitudinal studies are required to study the relationship between corneal nerve changes and other markers of severe disease phenotype.
